# pH dependency of the structural and photophysical properties of the atypical 2′,3-dihydroxyflavone†

**DOI:** 10.1039/d0ra06833k

**Published:** 2020-09-22

**Authors:** Luc Labarrière, Aurélien Moncomble, Jean-Paul Cornard

**Affiliations:** Univ. Lille, CNRS, UMR 8516 – LASIRE – Laboratoire avancé de spectroscopie pour les interactions, la réactivité et l'environnement F-59000 Lille France jean-paul.cornard@univ-lille.fr

## Abstract

2′,3-Dihydroxyflavone (2′3HF) is a natural flavonol that has barely ever been studied, however the scarce studies of its physico-chemical properties have highlighted its atypical behaviour. We present a structural and spectral study of 2′3HF, performed using UV-visible absorption and fluorescence spectroscopies, coupled with DFT and TD-DFT calculations. Although its structure is close to that of 3-hydroxyflavone, 2′3HF shows a much lower p*K*_a_ value. We show that the origin of this particularity is the substitution by a hydroxyl group on position 2′, that induces a stronger inter-ring interaction weakening the bonding of the proton at position 3. The main absorption band of the is red-shifted upon deprotonation. The remaining proton is highly bonded in between oxygen atoms 3 and 2′, making the second deprotonation unattainable in methanol. The neutral form can undergo an excited-state intramolecular proton transfer to emit dual fluorescence by the normal and tautomer forms. We suggested five geometries to be the sources of the emission bands, and showed that the energy barriers to interconversions were almost null. The anion is also fluorescent. The Stokes shifts for the neutral normal and anion species are extremely high, that can be explained by the conformational rearrangement, as the species go from twisted in the ground-state, to planar in the excited-state. Finally, another emission band is evidenced when exciting in the vicinity of the absorption maximum of the anion species in acidic medium. We suggest an aggregate with the solvent to be the origin of the emission.

## Abstract 2

The assignment of the multiple fluorescence emission wavelengths of 2′,3-dihydroxyflavone highlights its particular properties compared to analogues.

## Graphical abstract

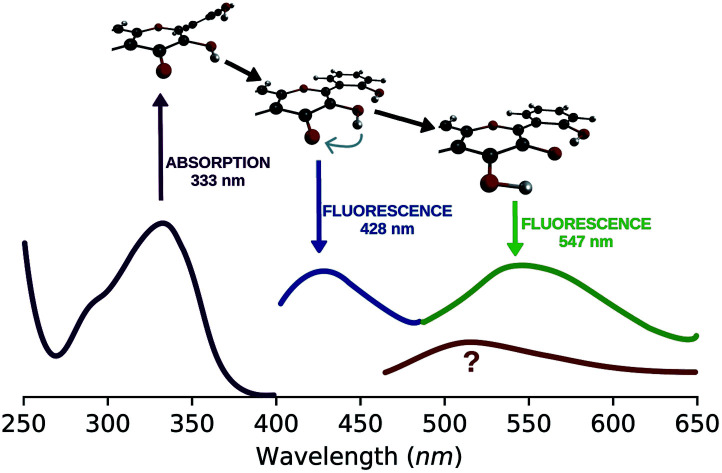

## 1. Introduction

Flavonoids are a class of phenolic natural compounds, widely found in plants as secondary metabolites.^1^ Their effect on human health is the most important field of research on their subject, as they have numerous biological applications, among which are neuroprotective,^2^ anticarcinogenic,^3–5^ antimicrobial,^6,7^ and antioxidant^8^ effects. Promising effects against Alzheimer's disease have been evidenced^9–11^ and flavonoids are thought to be responsible for the “French paradox”.^12,13^ They are also widely studied for their optical properties, as they could provide UV-protection^14,15^ and colour to plants.^16^

**Fig. 1:**
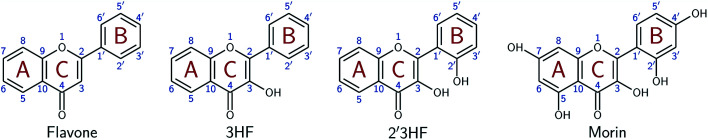
Structures of flavone, 3HF, 2′3HF and morin, with IUPAC atomic numbering and ring labelling.

This study focuses on 2′,3-dihydroxyflavone (2′3HF), a natural flavonol, subclass of flavonoids.^17^ The most simple flavonol is 3-hydroxyflavone (3HF) whose structure is depicted on Fig. 1, composed of a hydroxylated chromone moiety (A and C rings) and a phenyl (B ring).

The colour of 3HF depends on pH, as it is yellow in basic medium, and absorbs only UV light at a lower pH. It exhibits a dual fluorescence that was first described by M. Kasha and K. Sengupta,^18^ who attributed the violet fluorescence to the normal species, and the green one to the proton-transfered (PT) tautomer. This was confirmed later on, and the mechanism of the intramolecular proton transfer at the excited state (ESIPT) has been extensively studied ever since.^19–21^ The interesting photophysics of 3HF and its derivatives are highly valuable. Indeed, they are exploited as fluorescent probes in a wide variety of fields such as the chemistry of materials,^22,23^ quantitation of biomolecules^24,25^ and bioimaging.^26,27^

Very little is known however on 2′3HF. It has shown potent antiviral properties *in vitro* and *in vivo* against influenza A virus,^28^ and it is an inhibitor of HIV-1 proteinase.^29^ It protects cells against oxidative damage,^30,31^ and is currently studied for its ability to improve the preparation and isolation of stem cells for regenerative medicine applications.^32–34^ Its crystal structure has been characterized, using X-rays,^35^ and its mass spectrum recorded.^36^

2′3HF is a good candidate of study, as it is a simple 3HF derivative, but has shown to behave quite differently from other flavonols. Indeed, Porter and Markham^37^ suggested in 1970 the existence of a 2′3HF anion formation at unusually low pH, due to the strong O3H2′ hydrogen bond (HB), to explain the lack of a bathochromic shift of the long wavelength absorption band (band I) during Al^3+^ titration. Also, 2′3HF showed high unusual electrophoretic mobility^38^ and the same reason was evoked to explain this behaviour. More recently, Burns *et al.*^39^ recorded the ^1^H and ^13^C NMR spectra of multi-hydroxylated flavones, and established a method to predict a ^13^C NMR shielding using the spectrum of the flavone molecule (of structure depicted on Fig. 1). They showed that the chemical shifts of molecules having hydroxyl groups interacting with one another were not predicted accurately. 2′3HF was one of the molecules that showed this symptomatic behaviour.

This study aims at performing a comprehensive structural analysis of 2′3HF conformers, both in the ground and in the first excited states. For this purpose, throughout the paper, 2′3HF will often be compared to 3HF, but also to morin (Fig. 1), the most studied 2′-hydroxylated flavonol. This structural study is followed by a thorough analysis of 2′3HF optical properties: the absorption and fluorescence emission properties have been studied and are presented alongside.

## 2. Material and methods

### 2.1. Experimental details

#### 2.1.1. Reagents

2′3HF was purchased from Alfa Aesar. It is sparingly soluble in water and all the experiments were carried out in methanol solutions (ultrapure, spectrophotometric grade, 99.8%), also purchased from Alfa Aesar. 2′3HF concentrations were of 10^−5^ mol L^−1^ (4 × 10^−5^ mol L^−1^ for the titration experiment), with small additions of acid (HCl, from Fluka) and base (NaOH, white pellets, from Fischer Scientific) solutions. Water was obtained by a Millipore water purification system at 18 MΩ cm^−1^.

#### 2.1.2. Instrumentation

A Hanna pH meter was used for apparent pH measurements. UV-visible spectra were recorded with a double beam Cary 100 (Varian) spectrometer with a spectral resolution of 1 nm. The excitation and emission fluorescence spectra were recorded with a Fluorolog (Horiba) spectrofluorimeter with a resolution of 2 nm.

### 2.2. Computational details

The Gaussian 16 software^40^ was used to perform the time-independent and Time-Dependent Density Functional Theory (DFT and TD-DFT) calculations using, the PBE0 ^41,42^ hybrid functional along with Pople's 6-311+G(d,p) basis-set^43–46^ and the Polarizable Continuum Model (PCM)^47,48^ to simulate the solvent. The optimized geometries were confirmed as minima on the potential energy surface by analytically calculating the Hessian and verifying that no imaginary frequencies were obtained.

A relaxed energy scan was performed in the ground-state by fixing the inter-ring dihedral angle using a 5° step, and optimising all the other coordinates. This computation has been performed using 6-311++G(d,p) basis-set. The effect of adding a diffuse function to the basis-set was also tested on several other calculations, the differences (geometrical parameters and energies) were small enough to stick to the lower level of theory for most computations.

The location of transition structures was performed using, depending on the case, either the default Berny algorithm^49,50^ implemented in Gaussian 16, or the STQN method.^51^ This was systematically followed by an IRC calculation,^52^ verifying that the two obtained structures corresponded to the expected minima.

The electronic absorption spectra were reproduced by computing the required amount of excited-states using TD-DFT to fully describe the UV-visible domain experimentally studied. The solvent was once again described using PCM in a linear response non-equilibrium approach. The fluorescence emission energies were obtained by optimizing the first singlet excited-state, and computing the first transition energy, with the solvent equilibrated in the first excited-state.

Finally, several natural bond orbital (NBO) analyses^53,54^ were performed on the wave functions. Those analyses provided Wiberg bond indices^55^ corresponding to neighbouring pairs of atoms.

## 3. Results and discussions

### 3.1. Ground-state

#### 3.1.1. Neutral form's geometry

**Fig. 2:**
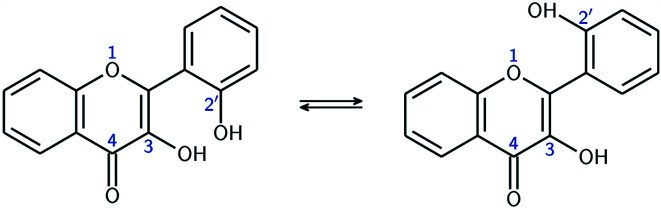
Equilibrium between the two conformers A (left), and B (right).

Compared to 3HF, 2′3HF exhibits an additional hydroxyl group at position 2′, a structural feature that allows the formation of a HB between O3 and H2′ giving a 7-membered ring, or O1 and H2′ giving a 6-membered ring. The two conformers, the first one with the O3H2′ HB noted A, and the second one with the O1H2′ HB noted B, are depicted on Fig. 2.

Both geometries were optimized in the gas phase and in methanol. The results are shown in Table 1, with energies referring to the chemical equation A ⇄ B. The differences in energy are higher in the gas phase than in methanol. This is not surprising as one would expect the intramolecular HBs to be weakened by interactions with the solvent, thus diminishing the effect of them on the overall potential energy.

**Table 1:** Computed differences in energy in kcal mol^−1^ between the A and B forms, in the gas phase and in methanol

	Δ_r_*E*°	Δ_r_*G*°
Gas	2.38	1.97
Methanol	1.88	1.59

**Fig. 3:**
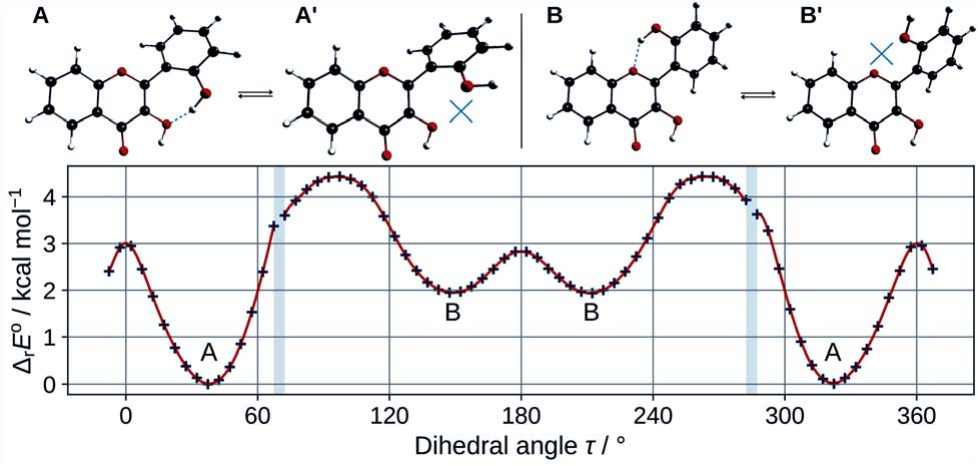
Optimized A, A′, B, and B′ conformers (top). Evolution of the molecular energy with the variation of the C3C2C1′C2′ dihedral angle (*τ*) (bottom).

In order to estimate the strength of the OXH2′ HBs, conformers with the O2′H2′ bond in the opposite direction were optimized in methanol. Their energies were compared to the ones of their corresponding most stable conformers according to the chemical equations A ⇄ A′ and B ⇄ B′, shown at the top of Fig. 3. The results are listed in Table 2.

**Table 2:** Computed differences in energy in kcal mol^−1^, for the removal of the OXH2′ HB in A and B

	Δ_r_*E*°	Δ_r_*G*°
A ⇄ A′	3.30	2.50
B ⇄ B′	1.27	0.47

These values must be interpreted with caution, as they do not exactly correspond to the energies of the HBs, since the other internal coordinates were allowed to relax and reduce the potential energy. Both HBs are weak, however the one in B is shown to be the weakest. This is also confirmed by observing the HB lengths, 1.710 Å and 1.879 Å, in A and B (knowing that they display a 6 and 7-membered ring) respectively. Taking into account the entropic term in the energy, the structures get even closer in energy. It is satisfactory to observe that both Δ_r_*E*° and Δ_r_*G*° are almost identical to those obtained for morin.^56^

The energy barrier of rotation of the B ring has been evaluated in methanol, by computing the energy of a set of geometries connecting A to B. The results are shown on the graph of Fig. 3. The energy profile shows discontinuities as artefacts generated during the relaxed scan. Those correspond mostly to energy jumps during the breaking of the O3H2′ HB. The activation energy required to rotate the B ring to convert A into B is 4.4 kcal mol^−1^. This is consistent with previous studies on flavones,^57^ that showed that flavonols had a higher torsional energy barrier than regular flavones. This is due to the position 3 hydroxyl group, that induces an inter-ring attractive interaction with position 2′. The potential energy minima appear at *τ* angles of 37.4° (A), and 148.6° (B).

The theoretical framework that we used cannot single out any species, as the energy difference between A and B is too small to be conclusive. However, A, the lowest energy conformer, is the one observed in the crystal phase.^35^ Moreover its computed electronic transitions are closest from the measured UV-visible absorption bands (see Section 3.1.3), so the structural analysis will focus on this particular conformer.

Some structural data are gathered in Table 3. The geometry does not change much when performing the calculation in methanol instead of in the gas phase. However, a few observations can be made. Indeed, the *τ* angle between the two moieties and the O4H3 HB length increase, indicating a weaker conjugation effect and HB.

**Table 3:** Main structural parameters of the 2′3HF in crystal (from the literature^35^), gas and methanol phases (A conformer). The structural parameters are also given for morin (from the literature^56^), and 3HF, computed at the same level of theory (in methanol). Bond lengths are given in Å, and valence and dihedral angles are given in °

	Distances						Angles				
	2′3HF			Morin	3HF		2′3HF			Morin	3HF
	Solid	Gas	MeOH				Solid	Gas	MeOH		
C2C3	1.361	1.359	1.360	1.361	1.366	C2C3C4	122.2	123.2	123.0	122.2	122.0
C3C4	1.439	1.447	1.445	1.437	1.450	C3C4C10	115.4	115.2	115.2	116.1	116.0
C5C6	1.369	1.378	1.378	1.382	1.377	C4C10C9	119.3	118.5	118.6	119.4	118.3
C6C7	1.397	1.402	1.403	1.401	1.404	C4C10C5	122.0	122.4	122.5	121.9	122.7
C7C8	1.369	1.380	1.381	1.392	1.380	C10C5C6	120.3	120.2	120.2	119.9	120.2
C8C9	1.397	1.395	1.395	1.384	1.396	C5C6C7	120.1	119.9	119.9	119.5	119.9
C5C10	1.405	1.402	1.404	1.417	1.404	C6C7C8	121.0	121.0	120.9	122.0	120.9
C4C10	1.453	1.451	1.450	1.428	1.447	C7C8C9	118.7	118.9	118.8	117.8	118.8
C9C10	1.390	1.397	1.398	1.402	1.397	C8C9C10	121.3	121.0	121.2	122.1	121.2
C2C1′	1.475	1.463	1.464	1.459	1.464	C8C9O1	116.8	116.7	116.6	117.3	116.8
C1′C2′	1.397	1.412	1.411	1.415	1.404	C9O1C2	120.4	121.7	121.6	122.3	122.3
C2′C3′	1.395	1.398	1.397	1.392	1.389	O1C2C3	120.8	119.2	119.5	119.4	119.4
C3′C4′	1.372	1.381	1.383	1.387	1.391	O1C2C1′	111.4	112.2	112.3	112.2	112.3
C4′C5′	1.382	1.394	1.395	1.398	1.392	C3C2C1′	127.8	128.6	128.2	128.4	128.4
C1′C6′	1.404	1.406	1.406	1.406	1.404	C2C1′C2′	123.9	123.8	123.8	124.4	122.1
C5′C6′	1.386	1.380	1.382	1.379	1.387	C1′C2′O2′	124.0	124.7	124.3	124.2	
C2O1	1.366	1.360	1.355	1.359	1.357	C2′O2′H2′	108.7	110.8	110.5	110.6	
C9O1	1.363	1.352	1.353	1.353	1.349	C3′C2′O2′	116.5	116.1	116.3	115.8	
C3O3	1.360	1.355	1.356	1.359	1.345	C1′C2′C3′	119.4	119.2	119.4	119.9	120.3
C4O4	1.243	1.232	1.237	1.254	1.238	C2′C3′C4′	121.1	121.2	121.1	120.9	120.7
C2′O2′	1.359	1.344	1.350	1.348		C3′C4′C5′	120.2	120.2	120.1	120.1	119.5
O3H2′	1.810	1.715	1.710	1.700		C4′C5′C6′	119.7	119.2	119.3	118.9	120.4
O2′H2′	0.892	0.973	0.975	0.976		C5′C6′C1′	120.9	121.9	121.8	122.5	120.7
O3H3	0.832	0.979	0.977	0.975	0.977	C6′C1′C2	117.4	117.7	117.7	118.0	119.4
O4H3	2.408	1.965	2.016	2.060	1.968	C10C4O4	123.0	126.2	126.0	124.6	125.6
						C3C4O4	121.7	118.6	118.8	119.3	118.4
						C4C3O3	119.1	114.0	115.0	115.7	114.3
						C2C3O3	118.7	122.8	122.0	122.1	123.7
						C3O3H3	113.7	103.7	104.8	105.6	103.8
						O4H3O3	106.0	119.7	117.7	116.5	119.8
						O2′H2′O3	155.8	157.4	156.5	157.3	
						C3C2C1′C2′ (*τ*)	42.8	36.2	37.4	35.8	1.1

The analysis of the X-ray data from the literature^35^ shows that 2′3HF forms dimers in the crystal phase. Indeed, the O4 and H3 of one 2′3HF molecule, bond themselves, to the H3 and O4 of another 2′3HF, respectively. The dimer is highly sterically constrained around positions 3, 4 and 2′, explaining why the *τ* and C3O3H3 angles are larger than in solution.

For comparison purposes, the structural parameters of 3HF, calculated at the same level of theory in methanol solution, and morin, taken from the literature,^56^ have been added in the table. 3HF is almost completely planar, in opposite with 2′3HF and morin; it can be noticed that the O4H3 HB length is shorter in 3HF. This fact can be explained by the presence of the O2′H2′ hydroxyl group in the two other flavonols that allows the formation of the O3H2′ intramolecular HB. The O3H2′ HB length is shorter than the O4H3 one in both 2′3HF and morin, and it is well known that the length of the bond is one of the important parameters in assessing the strength of a HB, so the H2′ appears more bonded to its HB-acceptor than H3. This structural feature, along with the fact that O3H3 and O2′H2′ distances are similar in all structures, would indicate that H3 is the most acidic proton of the two.

Another key structural parameter to understand the properties of flavonols is the length of the inter-ring bond C2C1′. However, the lengths are similar in the studied structures.

#### 3.1.2. Acid–base properties

Höfener *et al.* suggested, using a similar computational method as ours, a first deprotonation of morin at position 7.^58^ We find it surprising that they didn't consider deprotonations at positions 3 et 2′ however, and it was shown later on by our group that the 2′ hydroxyl group was responsible for the higher acidity of morin compared to other flavonols, yielding an easy removal of either protons 2′ or 3.^56^ Studying 2′3HF represents the simplest possible system in order to test this hypothesis and the structural analysis showed that it could share the same behaviour with morin.

**Fig. 4:**
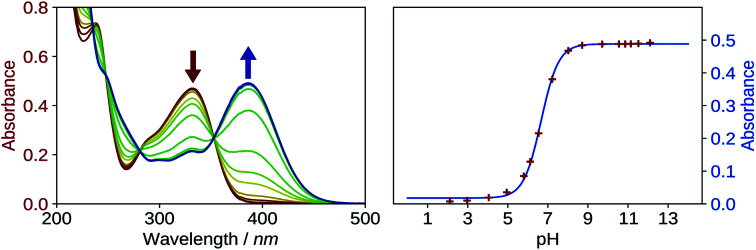
Evolution of the UV-visible absorption spectrum (left), along with the increase in pH, starting from 2 to 12, in methanol. Evolution of the absorbance at *λ*_abs_ = 387 nm, plotted against pH (right). The red points are experimental values whereas the blue line is the model.

The first p*K*_a_ of 2′3HF was obtained carrying out a titration against sodium hydroxide in methanol. Fig. 4 shows the evolution of the UV-visible absorption spectrum during the titration. Along with the increase in apparent pH, the band I (located at 333 nm in acidic medium) is red shifted to 387 nm, and several isosbestic points are simultaneously observed, indicating an equilibrium between two species. The overall spectrum shape is consistent with its description by Porter and Markham.^37^ On the same figure, the absorbance at 387 nm is plotted against pH, the curve being obtained by fitting the monoprotic acid–base sigmoid function, of equation
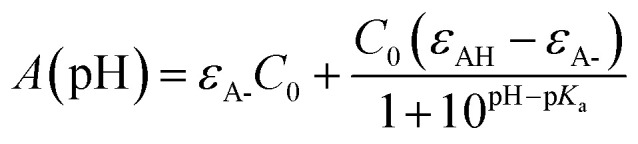
using the least squares method. *ε*_AH_ and *ε*_A-_ are the molar extinction coefficients of the acid and base species, respectively, and *C*_0_ is the total concentration of 2′3HF. The model yields an unusually strong acidity with a p*K*_a_ of 6.67. Indeed, a value of p*K*_a_ = 9.6 was found for 3HF in water,^59^ even though p*K*_a_ values are usually lower in water than in methanol. The p*K*_a_ value of 2′3HF is thus very low and almost the same as of morin.^56^

**Fig. 5:**
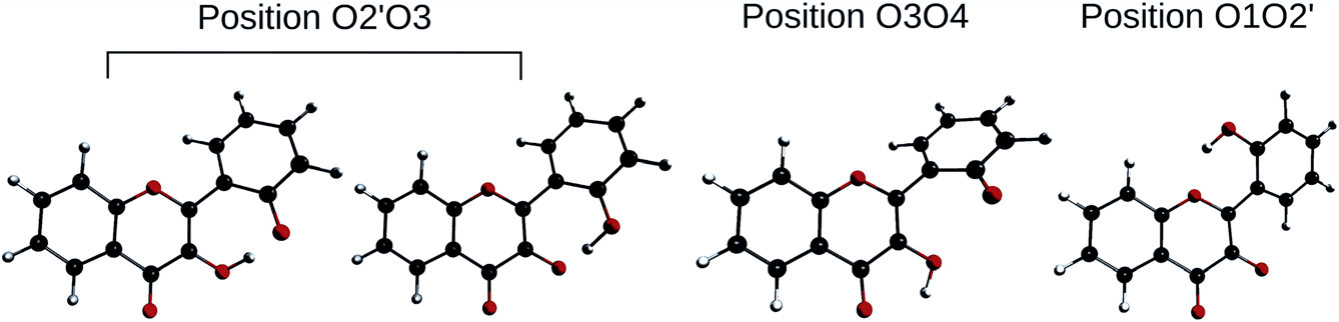
Optimized geometries depicting the possible positions for the remaining proton, after the removal of the one of the two.

In order to determine the geometry of the anion, several geometry optimizations were performed with initial structures built from A and B forms, by removing a proton on a hydroxyl group, with the remaining proton lying between O3 and O4, O2′ and O3, or O1 and O2′ (positions O3O4, O2′O3 and O1O2′). The optimized structures are represented on Fig. 5. Both positions O3O4 and O1O2′ yield energies at least 11 kcal mol^−1^ higher than position O2′O3. Two distinct minima are found for a geometry with the remaining proton lies between O2′ and O3, with a difference in energy of Δ_r_*E*° = + 0.09 kcal mol^−1^ in favour of the form with H2′ on O3. In order to check the existence of two individual species, the transition state (TS) between the two conformers was calculated. The results give an activation energy required to move the proton from O3 (dep2′), to O2′ (dep3) of Δ*E*^‡^ = + 0.13 kcal mol^−1^. The careful analysis of the imaginary frequency shows that it corresponds to the movement of H2′ from O3 to O2′, as expected. We must point out that dep2′ and dep3 appear very similar, both in energies and geometries. The prediction of the existence of two distinct species must be interpreted with caution as even the smallest adjustments in the theoretical model could yield only one stable conformer. We decided however to describe both species, as they display quite interesting differences in optical properties (see Section 3.1.3).

The main structural parameters of dep2′, dep3 and the TS are displayed in Table 4. The changes upon deprotonation appear mostly around C3 and C2′ as expected.

**Table 4:** Main structural parameters of dep2′, dep3 and of the TS between the two, in methanol. Bond lengths are given in Å, and valence and dihedral angles are given in °

	dep2′	dep3	TS
C2C3	1.379	1.386	1.383
C2C1′	1.463	1.465	1.464
C1′C2′	1.432	1.422	1.425
C3O3	1.330	1.305	1.315
C4O4	1.236	1.236	1.236
C2′O2′	1.302	1.328	1.317
O3H2′	1.082	1.305	1.205
O2′H2′	1.328	1.095	1.171
C1′C2′C3′	117.1	118.7	118.1
C2C3C4	120.5	118.9	119.5
C3O3H2′	106.0	105.3	105.7
C2′O2′H2′	106.0	106.8	106.6
O2′H2′O3	170.1	170.7	171.4
C3C2C1′C2′ (*τ*)	30.4	30.2	29.5

The *τ* angle between the two moieties is around 30°, a 7° lower value than in A, this is expected as the 3-2′ inter-ring interaction is higher in these forms, however the inter-ring bond C2C1′ is only slightly shortened.

In dep2′ and dep3, the HB lengths are respectively 1.328 and 1.305 Å. Those distances are very low, and the O2′H2′O3 angles, of respectively 170.1° and 170.7°, are close to the linear limit. Those values indicate a very strong bonding of the proton, and a predicted high second p*K*_a_ value, explaining no second deprotonation has been observed using sodium hydroxide.

#### 3.1.3. Electronic excitation energies

A comparison of the UV-visible spectra of the neutral and deprotonated species of 2′3HF with the calculated electronic transitions was made (i) to confirm the results of the structural study, and (ii) to give a complete assignment of the different spectral bands experimentally observed.

**Fig. 6:**
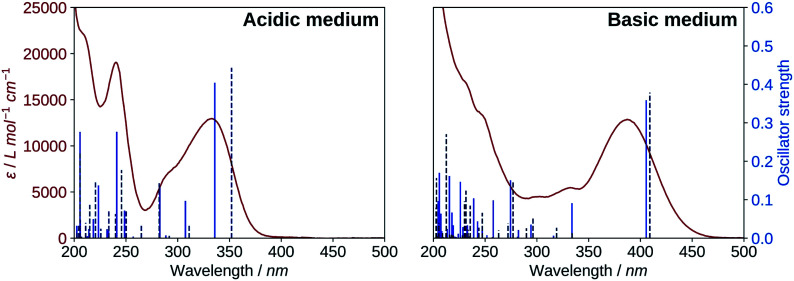
Comparison of the UV-visible absorption spectrum (red) of 2′3HF with the computed electronic transitions (blue) for the proposed species. Left: spectrum in methanol + HCl medium and electronic transitions of A (solid) and B (dashed). Right: spectrum in methanol + NaOH medium and electronic transitions of dep2′ (solid) and dep3 (dashed).

The results for the protonated species are shown on the left of Fig. 6. The transitions computed for A reproduce well the absorption spectrum, whereas B yields a too low first excitation energy. Overall, the spectrum is better reproduced from the electronic transitions of A. 2′3HF exhibits the usual optical behaviour of flavonols, with absorption of UV radiations at 240 nm (band II) and 333 nm (band I), the latter having a shoulder at 290 nm. It can be noticed that this shoulder is well reproduced by the calculations and corresponds to two electronic transitions. Few descriptions of the UV-visible absorption spectrum of 2′3HF are found in the literature,^37,60^ however, they are consistent with the one that we observe. The spectrum is reported to be red shifted in ethanol, with band I at 353 nm ^61^ (340 nm according to Hayashi *et al.*^62^), with an overall shape close to the one we get.

The electronic transitions computed for the deprotonated species, dep2′ and dep3 (Fig. 6, right), show a great similitude both in energy and oscillator strength. Compared to the experiment, the computed first excitation energies of dep2′ and dep3 are red-shifted by 0.15 eV (19 nm) and 0.17 eV (22 nm), respectively, compared to the maximum of band I. These differences are below TD-DFT accuracy.

**Fig. 7:**
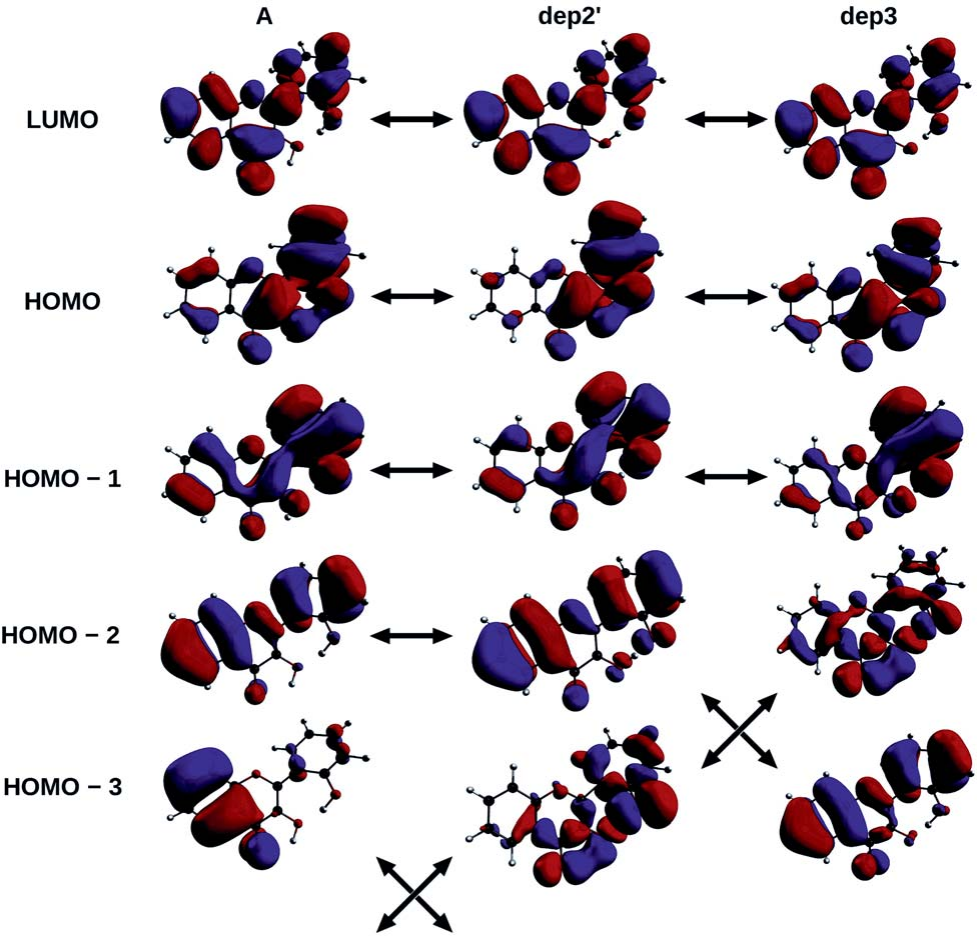
HOMO – 3, 2, 1, HOMO and LUMO of A, dep2′, and dep3 species, The arrows indicate the similarities of the MOs between the different structures.

The informations on the first transitions of low energy are gathered in Table 5 for the three species A, dep2′ and dep3. The first transition is a HOMO→LUMO. Looking at the involved orbitals, depicted on Fig. 7, one can see that these transitions have a ππ* and charge-transfer (CT) character from the phenol to the chromone moiety. This CT behaviour is greatest in dep2′. Analysing this transition for A shows that electronic density is removed from O3 and added onto O4. This mechanism is known for allowing ESIPTs in 3HF derivatives,^63^ as the acidity of H3 and the basicity of O4 are increased. It represents a first indication that an ESIPT could occur in 2′3HF. This possibility is discussed with more details in the next section.

**Table 5:** Computed first important transition energies in nm (eV) and oscillator strengths for A, dep2′, and dep3. H stands for HOMO, and L for LUMO

	*E*	*f*	Nature
A	336 (3.69)	0.402	H → L (95%)
	307 (4.03)	0.095	H – 1 → L (93%)
	283 (4.39)	0.132	H – 2 → L (83%)
dep2′	405 (3.06)	0.357	H → L (98%)
	334 (3.71)	0.089	H – 1 → L (96%)
dep3	409 (3.03)	0.379	H → L (98%)
	334 (3.71)	0.014	H – 1 → L (48%)
			H – 2 → L (40%)

The second transition is mainly a HOMO – 1 → LUMO one in all cases, with low probability of transition. However, dep3 behaves somewhat differently. Indeed, although it has the same transition energy as dep2′, the transition in dep3 has a 40% contribution coming from the HOMO – 2 → LUMO. This contribution has a strong nπ* character, resulting in an even lower oscillator strength due to the selection rules and, thus, in a large change on the absorption spectrum. To understand this difference, one must notice that the energy levels corresponding to HOMO – 3 and HOMO – 2 are swapped between the two species.

### 3.2. Excited-state

#### 3.2.1. Fluorescence emission in acidic medium

**Fig. 8:**
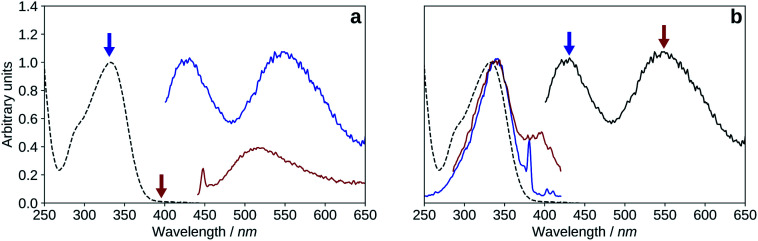
Normalized UV-visible absorption (black, dashed) spectrum compared to the fluorescence emission and excitation spectra of 2′3HF in methanol + HCl medium. (a): *λ*_exc_ = 335 nm (blue), *λ*_exc_ = 395 nm (red). (b): *λ*_exc_ = 335 nm (black), *λ*_em_ = 430 nm (blue), *λ*_em_ = 550 nm (red).

The fluorescence emission spectrum of 2′3HF in methanol (in acidic medium), and the fluorescence excitation spectra compared to the absorption spectrum are shown on Fig. 8. 2′3HF exhibits a dual fluorescence, and doing an analogy with some other 3HF derivatives, the normal form would emit at 428 nm (band N), and the tautomeric form at 547 nm (band T). This hypothesis is corroborated by the fact that the fluorescence excitation spectra corresponding to the two emission maxima are comparable, even though band T appears to also have a contribution from the excitation of another species, absorbing around 390 nm. Indeed, another emission band located at 517 nm is evidenced using a 395 nm excitation wavelength. The origin of this band is discussed in Section 3.2.4. On the excitation spectrum at 430 nm emission, a sharp peak can be seen at 381 nm, along with two little humps at 404 and 410 nm. Those are due to Stokes Raman scattering from methanol, and correspond respectively to C–H stretching (∼3000 cm^−1^), CH_3_ deformation (∼1500 cm^−1^), and C–O stretching (∼1050 cm^−1^).^64^

The Stokes shifts for the N and T bands are of 95 nm (6666 cm^−1^) and 214 nm (11 748 cm^−1^), respectively. Both Stokes shifts are very large, and the second one is typical of an ESIPT, where large geometry and electronic changes occur.

The N/T band ratio was around 1 in all of our experiments, however, it seemed to be fluctuating. This fluctuation was attributed to the fact that the solvent used contained traces of water, with an uncontrolled H_2_O/MeOH ratio. Moreover, for some experiments, aqueous HCl (or NaOH) solutions were added in small amounts.

By optimizing the geometry of the first singlet excited-state, two local minima were found. They correspond to the normal form A* with the keto group lying at position 4, and a tautomeric form, noted T3* (keto group at position 3). By modifying the initial geometry, another minimum was found, with the keto group at position 2′, noted T2′*. Finally, two other geometries originating from the form B were found: B* and BT3* (the latter having the keto group at position 3, and position 2′ in front of position 1).

**Fig. 9:**
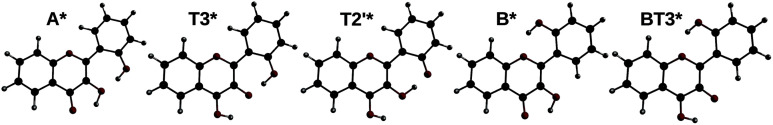
A*, T3*, T2′*, B* and BT3* optimized geometries.

All five geometries are depicted on Fig. 9, and the relative energies and computed emission wavelengths are gathered in Table 6. All tautomer geometries have lower electronic and Gibbs free energies than their normal counterpart in the S1 state, meaning that the PT could occur if it is fast enough.

**Table 6:** Computed electronic and Gibbs free energies (kcal mol^−1^) between the five geometries in the S1 state relative to the most stable species, and their computed emission wavelength (nm). The differences between the theoretical wavelengths and their corresponding experimental values (either band N or T, depending of the species) are shown between brackets, in eV

	Δ_r_*E*°	Δ_r_*G*°	*λ*_em,theo_ (*vs.* exp)
A*	14.69	14.49	430 (0.01)
B*	14.52	14.81	431 (0.02)
T3*	0.00	0.54	556 (0.04)
T2′*	0.35	0.00	588 (0.16)
BT3*	7.56	8.21	563 (0.06)

Among the two species that can explain the band N, A* and B* forms have almost the same energy and emission wavelength. It is interesting to note however that the electronic term of the energy (and the entropic correction) favours B*, whereas the vibrational correction favours A*, reversing the result.

The band T is also very well described by all 3 tautomers, as even T2′* shows an emission energy that deviates from the experiment by only 0.16 eV, below TD-DFT accuracy. Here again, T3* and T2′* are so close in energy, that T3* is more stable when looking at the electronic energy, whereas T2′* is favoured after vibrational and entropic corrections.

The theoretical study being based on the absorption and emission spectra, it will be nearly impossible to confirm the existence or non-existence of any of those species. However, for the same reasons we stated in Section 3.1.1, this work will focus on A*, T3* and T2′*.

In order to estimate if the PT is feasible within the timescale of fluorescence, we explored the potential energy surface, searching for TSs. Two were located: one between A* and T3* (noted TS:A*-T3*, Δ*E*^‡^ = + 0.6 kcal mol^−1^) and the other between T3* and T2′* (noted TS:T3*-T2′*, Δ*E*^‡^ = + 0.1 kcal mol^−1^). However, attempting to find a TS between A* and T2′* yielded TS:A*-T3* instead. This leads to the conclusion that intra-molecular PTs to get T3*, or even double intra-molecular PTs to get T2′*, are extremely fast, with almost null energy barrier.

Both experiments and calculations showed that 2′3HF exhibits a dual fluorescence, induced by an ESIPT, with an N/T band ratio remarkably close to 1. However, the purpose of this study is not to describe the PT mechanism. As a matter of fact, only the intra-molecular PT is shown to be feasible here, and the description of a solvent assisted PT, for example, would require explicit treatment of solvent molecules in high level excited-state molecular dynamics, far beyond the scope of this work.

#### 3.2.2. Neutral excited-state geometries

The main structural parameters of A* are displayed in Table 7. The main difference between A and A* is that A* is completely planar in the excited-state, and this structural difference could explain large Stokes shift of 6666 cm^−1^ already mentioned. Although most angles and bond lengths are kept the same, the O3H2′ HB gives a 7-membered ring planar, with significantly shorter HB distances compared to A, of 1.741 and 1.545 Å for O4H3 and O3H2′, respectively. The angles are also highly changed, and O2′H2O3 is almost linear.

**Table 7:** Main structural parameters, Wiberg indices, and NPA charges of A, A*, T3*, and T2′* calculated in methanol. Bond lengths are given in Å, and valence and dihedral angles are given in °

	A	A*	T3*	T2′*
**Distances (Wiberg indices)**				
C2C3	1.360 (1.53)	1.397 (1.29)	1.423 (1.22)	1.395 (1.35)
C3C4	1.445 (1.11)	1.441 (1.12)	1.425 (1.18)	1.416 (1.21)
C5C6	1.378 (1.52)	1.386 (1.46)	1.382 (1.49)	1.381 (1.50)
C6C7	1.403 (1.36)	1.394 (1.41)	1.398 (1.39)	1.400 (1.38)
C7C8	1.381 (1.49)	1.398 (1.39)	1.394 (1.40)	1.390 (1.43)
C8C9	1.395 (1.34)	1.383 (1.42)	1.384 (1.41)	1.387 (1.39)
C5C10	1.404 (1.31)	1.408 (1.29)	1.414 (1.26)	1.413 (1.27)
C4C10	1.450 (1.09)	1.427 (1.14)	1.402 (1.24)	1.405 (1.23)
C9C10	1.398 (1.32)	1.409 (1.27)	1.413 (1.23)	1.412 (1.24)
C2C1′	1.464 (1.07)	1.429 (1.23)	1.430 (1.24)	1.460 (1.12)
C1′C2′	1.411 (1.31)	1.457 (1.14)	1.451 (1.16)	1.456 (1.13)
C2′C3′	1.397 (1.36)	1.402 (1.33)	1.404 (1.33)	1.421 (1.25)
C3′C4′	1.383 (1.47)	1.377 (1.48)	1.379 (1.47)	1.382 (1.46)
C4′C5′	1.395 (1.39)	1.407 (1.30)	1.404 (1.32)	1.394 (1.38)
C1′C6′	1.406 (1.34)	1.420 (1.28)	1.420 (1.28)	1.392 (1.43)
C5′C6′	1.382 (1.48)	1.378 (1.49)	1.379 (1.49)	1.402 (1.34)
C2O1	1.355 (1.00)	1.375 (0.96)	1.376 (0.96)	1.376 (0.96)
C9O1	1.353 (1.00)	1.368 (0.97)	1.355 (0.99)	1.350 (1.01)
C3O3	1.356 (1.01)	1.331 (1.07)	1.287 (1.27)	1.321 (1.12)
C4O4	1.237 (1.57)	1.269 (1.38)	1.331 (1.10)	1.335 (1.09)
C2′O2′	1.350 (1.05)	1.319 (1.16)	1.313 (1.18)	1.282 (1.32)
O2′H2′	0.975 (0.68)	0.986 (0.64)	1.043 (0.54)	1.319 (0.26)
O3H3	0.977 (0.68)	1.007 (0.60)	2.017	2.034
O4H3	2.016	1.741	0.974 (0.70)	0.971 (0.71)
O3H2′	1.710	1.545	1.371 (0.21)	1.073 (0.49)

**Angles**				
C3C4O4	118.8	115.7	117.7	118.8
C4C3O3	115.0	110.9	115.5	114.1
C3O3H3	104.8	101.6	84.5	84.9
O4H3O3	117.7	125.9	116.3	115.1
O2′H2′O3	156.5	166.9	173.6	172.3
C3C2C1′C2′ (*τ*)	37.4	0.3	0.0	0.0

**NPA charges**				
C3	0.170	0.291	0.293	0.262
C4	0.459	0.328	0.321	0.323
C2′	0.346	0.403	0.390	0.398
O4	−0.660	−0.733	−0.644	−0.650
O3	−0.705	−0.667	−0.679	−0.705
O2′	−0.706	−0.613	−0.644	−0.650
H3	0.519	0.532	0.511	0.511
H2′	0.504	0.510	0.487	0.488

In order to check whether the geometry change (twisted to planar) is solely responsible for the high Stokes shift, one can try substituting the B ring on position 2′ with a group that does not modify significantly the electronic density, and to compare the obtained Stokes shift with the one observed for 3HF. This has actually already been done in the past. Indeed, Strandjord *et al.*^65^ methylated 3HF on multiple positions, including position 2′, and measured the Stokes shifts. The methyl group induces a minor electron donation effect, while generating a strong steric hindrance. This way, comparing 3HF, 2′-Me-3HF and 4′-Me-3HF, gives insights on the effect of the steric hindrance on the Stokes shift. The results are gathered in Table 8.

**Table 8:** Absorption and emission energies, and Stokes shift, in cm^−1^, of some 3HF derivatives

	*ν*_abs_	*ν*_em_	*ν*_SS_
3HF^65^	29 150	24 630	4520
2′-Me-3HF^65^	30 580	24 752	5828
2′3HF (this work)	30 030	23 364	6666
4′-Me-3HF^65^	28 730	24 390	4340
4′-OMe-3HF^66^	28 409	23 364	5045

Comparing 3HF and 4′-Me-3HF, one can see that the methyl substitution does not change much the Stokes shift, that reduces by only 180 cm^−1^, due to a combination of lowering both the excitation and emission energies. However, when the substitution happens on position 2′, the Stokes shift increases by 1308 cm^−1^. The substitution does not alter much the emission, but has a remarkable effect on the excitation energy. In the table, we also added the energies related to the substitution by a methoxyl group^66^ on position 4′. It shows that the substitution by an electron donor group (stronger than a methyl), reduces the emission energy more than the excitation energy. The Stokes shift can then be explained by a combination of a steric hindrance between positions 2′ and 3 (that generates large geometry changes upon excitation), and an electron donation effect on ring B.

The analysis of the HOMO → LUMO transition of A showed that part of the electronic density was moved from O3 to O4. In order to quantify the effect of electronic density reorganisation, we performed NBO and NPA (natural population) analyses. The values are gathered in Table 7.

The NBO analysis corroborates the observation, and the Wiberg indices are overall negatively correlated with bond lengths as expected. Indeed, the Wiberg index decreases from 1.57 to 1.38 for C4O4, increases from 1.087 to 1.142 for C4C10, and the natural charge on O4 decreases from −0.66 to −0.73, going from A to A*. This indicates that C4O4 becomes closer to an enolate, the whole group acting as a better base than in the ground-state. The opposite behaviour is observed for C3O3, and even more for C2O2. Indeed, their Wiberg indices and the charges on the oxygen atoms increase, whereas the C2C3 and C1′C2′ indices decrease. Note that O3H3 and O2′H2′ bond orders also decrease of 0.08 and 0.04 respectively, and the hydrogen atoms appear less bonded to their oxygens. Finally, C2C1′ is close to a single bond (1.07), that can easily rotate in the ground-state, whereas its order increases (1.23) in the excited-state. This behaviour is consistent with the molecule going from twisted to planar upon excitation.

Both tautomers are also completely planar. Their main structural parameters are gathered in Table 7. Along with the O2′–H2′–O3–H3–O4 network, the B and C rings are modified. The Wiberg indices confirm the positions of the keto groups that shift during A* to T3* conversion (C4O4 index evolves from 1.38 to 1.10 and C3O3 from 1.07 to 1.27) and during T3* to T2′* conversion (C3O3 index evolves from 1.27 to 1.12 and C2′O2 from 1.18 to 1.32).

#### 3.2.3. Fluorescence emission in basic medium

**Fig. 10:**
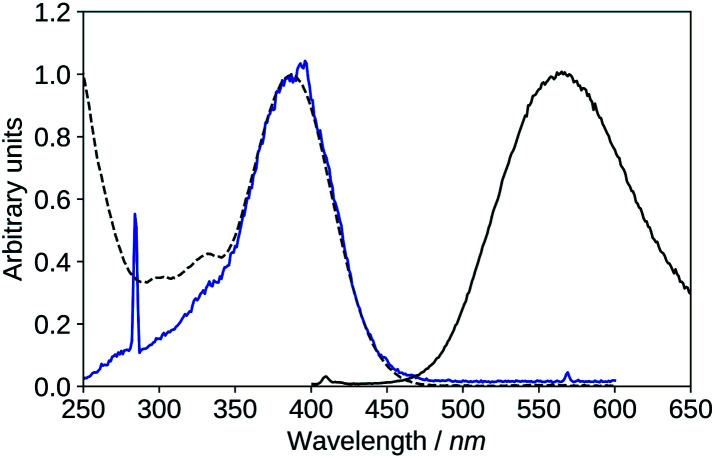
Absorption (black, dashed), excitation (blue, *λ*_em_ = 570 nm), emission (black, solid, *λ*_exc_ = 395 nm), spectra of 2′3HF in basic medium.

The fluorescence emission and excitation spectra of 2′3HF in basic methanol medium are depicted on Fig. 10, compared to the absorption spectrum. The excitation spectrum matches exactly the absorption spectrum indicating that the emission originates from an excited species formed through relaxation after exciting dep2′ or dep3 (noted dep in the remaining part of the paper).

The maximum of absorption of the anion is located at 387 nm, and the emission is maximum at 566 nm (noted band A). The sharp peak at 285 nm on the excitation spectrum is due to the second order Rayleigh scattering. The measured Stokes shift (8172 nm^−1^), even larger than the one observed for the normal neutral form.

In order to determine the geometry of the excited-state deprotonated species, we performed optimisations in the same way as for the ground-state, the remaining proton being moved to the previously suggested positions. Once again, the lowest energy is found when the proton lies at position O2′O3, however this time, only one conformer could be found, with the proton lying closer to the O2′ atom and a fully-planar geometry. The latter geometry will be noted dep* in the remaining part of the paper. The second possible conformer is less stable by Δ_r_*G*° = 5.78 kcal mol^−1^, and corresponds to the proton lying between O4 and O3, bonded to O3. Note that we also optimized a tautomer with the proton bonded to O4, that showed to be even less stable by Δ_r_*G*° = 1.32 kcal mol^−1^.

The same kind of explanation as for the neutral species can be applied to interpret the large Stokes shift. It is however less convincing than for A, as the *τ* angle goes from 30.4° in the ground-state (dep2′), to 0° in dep*, meaning that geometrical rearrangements happen to a lesser extent than in A.

The calculated emission wavelength for the most stable anion is 539 nm, 0.11 eV higher than the experimental value. This is a satisfactory result, as the accurate description of an anion is far more challenging than that of a neutral species, especially in the excited-state. In the end, the predicted Stokes shift is of 6099 cm^−1^ (using dep2′ as the ground-state species).

#### 3.2.4. Hypothesis on the 517 nm emission band

In Section 3.2.1, we showed that the emission band of the neutral 2′3HF observed at 550 nm contains a contribution from another species, evidenced by exciting in the vicinity of the absorption maximum of the deprotonated form of 2′3HF (in acidic medium). The spectrum is shown on Fig. 8(a). The band is expansive, and is maximum at 517 nm (noted band X). Several structural hypotheses have been made to explain the origin of this emission band,

##### Hypothesis 1: emission from a cation

Protonated flavonols have not been much investigated, and one would expect the protonation to blue shift the electronic transitions with respect to the neutral form. However, it was reported that the addition of H_2_SO_4_ red-shifts the absorption maximum of 3HF from 344 nm to 378 nm, and its normal fluorescence from around 410 nm to 430 nm.^67^ This behaviour was also found in other mono-hydroxylated flavonoids^59^ as, for example, 2′-hydroxyflavone shows emission at 514 nm upon protonation of its carbonyl group.

We optimized, then calculated the electronic excitation and emission energies of two different cation geometries, one with the HB network directed towards the keto-group (noted C4), and the other, with the HB network towards the 2′ hydroxyl group (noted C2′) in both the ground and excited states. The results are reported in Table 9.

**Table 9:** Computed relative free energies for the ground and first singlet excited states of geometries (left) C2′ and C4 (right) (in kcal mol^−1^), and absorption and emission wavelengths (in nm). The values reported in parenthesis are the differences with the experimental values of 390 nm (approximate excitation maximum), and 517 nm (emission wavelength) in eV. The depicted geometries are the conformers in their ground-state

	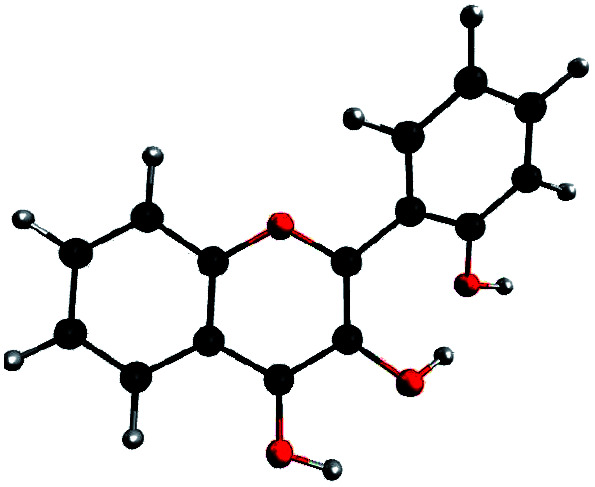	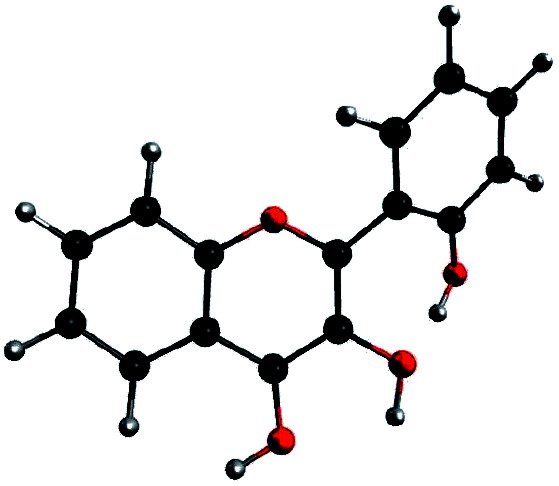
Δ_r_*G*° (S0)	0.00	5.63
*λ*_abs, calc_	373 (0.14)	422 (0.24)
Δ_r_*G*° (S1)	65.26	63.39
*λ*_em, calc_	457 (0.31)	527 (0.05)

The model predicts that the most stable cation species is C2′ in the ground-state, with a maximum of absorption at 373 nm, and a difference of 0.14 eV from the experimental 390 nm excitation value. In the excited-state, the HB network changes direction, as C4* is more stable than C2′*, and C4* emits a 527 nm fluorescence. The difference with the experimental band X maximum is 0.05 eV, and is thus very close.

This hypothesis is promising, as the calculated absorption and emission energies match the experimental values within TD-DFT accuracy. However, some experimental observations lead us to doubt this explanation. Indeed, the experimental data suggest that band X shows no pH dependence, or worse, a possible increase of intensity with increasing basicity. In any case, bands N, T, X and A can simultaneously be observed from the same solution, by varying the excitation wavelength, and the simultaneous observation of the cation, neutral and anion species is unlikely. In the end, the two computed species C4 and C2′ could probably exist in extremely acidic conditions, but we doubt they are responsible for the recording of band X. Experimenting in those conditions and observing the effects on band X could help clarify its behaviour with changes in pH.

##### Hypothesis 2: deprotonation of the anion for explaining band A

We already mentioned that the excitation spectrum of the emitting species of band A matched very closely the absorption spectrum of dep. This only ensures us that the excited species is dep, but does not provide any information on the geometry of the emitting species, apart from the fact that it is formed through relaxation from dep. We also mentioned that band X seemed to be increasing during the titration with NaOH, even though band A is so wide, that this observation can also be due to its simultaneously increasing intensity.

If the intensity of band X truly increases with pH, before being hidden by band A, one explanation can be that dep loses its remaining proton after excitation. Actually, the computed wavelength for dep* (539 nm) matches also the 517 nm maximum of band X, giving an even lower error of 0.10 eV, and a Stokes shift of 6497 cm^−1^, a shift better explained by the structural analysis made on A (in Section 3.2.2). Band A would thus correspond to the doubly deprotonated 2′3HF (dep2′3*). The calculation of the emission wavelength of the doubly deprotonated species yields 618 nm, with an error of 0.18 eV, below TD-DFT accuracy.

No second deprotonation can be seen in the ground-state, meaning that for this hypothesis to be true, the second p*K*_a_ must be much lower in the excited-state than in the ground-state. Calculating p*K*_a_ values using first principle methods is challenging. However, one can compute relative values that will be more accurate due to error cancellations. Indeed, subtracting both acid–base equilibria yields:2AH^−^ ⇄ A^2−^ + AH_2_
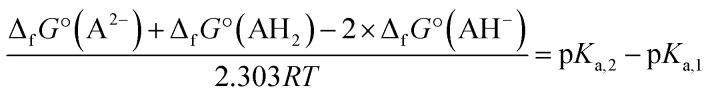


Using the calculated Gibbs free energies of A, dep2′ and dep2′3 for AH_2_, AH^−^ and AH^2−^, respectively in the ground-state, and A*, dep*, and dep2′3* in the excited-state, the second deprotonations are predicted to happen 24 and 28 units of pH after the first ones, for the ground and first excited states, respectively. While p*K*_a,2_ appears high in the ground-state, which is a result we already predicted, it is calculated to be even higher in the excited-state. This is also expected, as the molecule goes from twisted to planar from dep to dep*, tightening the bonding of H2′ to the oxygen atoms.

Based on this explanation, it appears chemically speaking doubtful that the band A would originate from dep2′3*, and in the same way, band X from dep*. Studies in various solvents, with various bases could help clarify this point, as a second deprotonation would be even more unlikely in hydrocarbon solvents. Until clarification of the behaviour of band A in such solvents, we favour the hypothesis of an emission from dep*, meaning that another explanation has to be found for explaining band X.

##### Hypothesis 3: formation of a complex with the solvent

The formation of a complex with the solvent has been suspected for a while in the case of 3HF. Dereka *et al.*^68^ showed that the long-wavelength absorption band of 3HF observed in neutral and basic media originated from two distinct species. The first one has been confirmed by this group, using infrared transient absorption, as the anion of 3HF, with a short lifetime of 40 ps (in methanol), pre-dominant in basic media. The other species has a longer lifetime of 2 ns, and is suspected to be a 3HF–solvent complex.

The emission of this species is located between bands N and T on the spectrum, very close to the anion one. If the same phenomenon is observed in the case of study, the situation would be different. Indeed, both species would absorb around the same energies (387–390 nm), but would emit at very distinct wavelengths of 517 nm (for the hypothetical solvent complex) and 566 nm (for the lone anion).

The nature of the long-wavelength long-lived species of 3HF is still unknown, and its spectral resemblance with the anion makes them often considered to be close in nature, or even confused. It is suspected that 3HF forms a stable complex with the solvent, of red-shifted absorption energy compared to the lone neutral 3HF, explaining the high dependence on the medium of the emission.^69–71^ Most research groups concluded however on the emission from the anion after an ESPT (intermolecular) to the solvent.^72–75^

The situation is more complicated in the case of 2′3HF, as band X and A are very different in intensities, energies and shape, a result that cannot be explained currently.

To confirm this hypothesis would require to improve the description of the solvent. Indeed, the addition of few explicit solvent molecules in addition to the PCM has shown to improve the accuracy in several cases, including for describing metal-complexes, and has been extensively used in our group.^76–79^ In this case, some attempts of adding explicit water or methanol molecules in the vicinity of O4, O3 and O2′ had very little effect on the excitation and emission energies. Thus, an accurate description of 2′3HF-solvent specific interactions would require statistical considerations, with the computation of many conformations in order to be meaningful, far beyond the scope of this study.

Finally, reproducing the study performed by Dereka *et al.*,^68^ involving time-resolved spectroscopic methods could be of great help. In particular, we suggest to check whether emission A corresponds to more than one species, and, if it is the case, if the 517 nm emitting species displays a similar lifetime to one of them.

## 4. Conclusions

A barely studied 3HF derivative, 2′3HF, has been investigated using a combination of electronic spectroscopies and density functional theory methods. 2′3HF has shown to exhibit the typical spectral behaviour of some flavonols, with an absorption band at 333 nm (band I), and a second one (band II) at 240 nm. It exhibits a dual fluorescence from the normal form (428 nm) and a tautomer (547 nm), analogously to 3HF, and shows a remarkably high Stokes shift from its normal form. A thorough analysis showed that the shift was mostly due to the steric hindrance due to the 2′-hydroxyl group. The PBE0/6-311+G(d,p) theoretical framework handled accurately the description of the absorption, and emission spectra of this flavonol. The ESIPT was properly predicted by the calculation of the relative energies between the conformers in the excited-state, along with the estimation of the energy barriers.

2′3HF exhibits a very low p*K*_a_ of 6.67, a particularity shared with morin, another 2′-hydroxylated flavonol. The band I is red-shifted to 387 nm upon deprotonation. The electronic excitation energies of the predicted most stable conformer of the anion reproduce well the absorption spectrum, even though the first excitation energy is a little under-estimated. The emission from the anion has been attributed to the 566 nm band, as the excitation spectrum matches closely the absorption spectrum.

Finally, we observed another emitting species, evidenced when exciting in the vicinity of the absorption maximum of the anion. Its emission maximum wavelength is 517 nm, and the band is best seen in acidic medium, even though it is possible that it is still present in basic medium, but hidden by the wide 566 nm band. We discussed three hypotheses on the origin of this band, and consider the hypothesis of an aggregate with solvent molecules to be favoured.

If we compare the results obtained on the studied chemical system with those already published on the very close derivatives, 3HF and morin, several significant facts can be highlighted.

The results obtained on morin raised questions; this is precisely the reason that led us to study a slightly simpler system focusing only on the substitution in position 2′ and 3. The major change and not the least, the fully protonated form of morin does not emit any fluorescence, whereas whatever the protonation state of 2′3HF, fluorescence emissions are detected. If we observe the molecular orbitals involved in the electronic transitions, they are however very similar. Once again this shows that the substitution pattern is paramount in the physico-chemical properties of these compounds.

The observation of a dual fluorescence of the fully protonated form is not new, this has been largely studied for 3-hydroxyflavone or quercetin, except that in the case of 2′3HF, the transfer of proton can take place from the 2′ position to the keto function, *via* the hydroxyl in position 3. This has never been considered for this substitution and it is a totally novel result.

Finally, for 3-hydroxyflavone, several studies have hypothesized a solvated form with different spectral properties without ever experimentally demonstrating it. In this paper, we believe that we have demonstrated the existence of this particular form by the observation of a fluorescence band at 517 nm by exciting in wavelengths longer than the absorption of the protonated molecule. We have considered several hypotheses for the molecular shape corresponding to this emission and the one retained was also a solvent complex. Some additional experimental and computational work that could shed light on them were proposed to confirm this hypothesis.

## Conflicts of interest

There are no conflicts to declare.

## Supplementary Material

RA-010-D0RA06833K-s001
