# Supplementary material for: pH dependency of the structural and photophysical properties of the atypical 2′,3-dihydroxyflavone
Source: RSC Adv. 2020 Sep 22;10(58):35017–30. doi: 10.1039/d0ra06833k (PMC9056863; doi:10.1039/d0ra06833k)
Supplement: RA-010-D0RA06833K-s001 [file RA-010-D0RA06833K-s001.pdf]

Electronic Supplementary Material (ESI) for RSC Advances.
This journal is © The Royal Society of Chemistry 2020

pH dependency of the structural and photophysical properties of the atypical 2',3-dihydroxyflavone

Luc Labarrière, Aurélien Moncomble, Jean-Paul Cornard

Univ. Lille, CNRS, UMR 8516 - LASIRE - Laboratoire avancé de spectroscopie pour les interactions, la réactivité et l'environnement, F-59000 Lille, France

Cartesien coordinates of atoms for the different forms of

2’,3-dihydroxyflavone

Table of contents

Ground-state optimized geometries 3

1. A....................................3
2. A’ 4
3. B 5
4. B’....................................6
5. dep2’....................................7
6. dep3....................................8
7. C2’....................................9
8. C4 10

Excited-state optimized geometries 11

1. A*....................................11
2. B* 12
3. T2’* 13
4. T3* 14
5. RT3*....................................15
6. dep* 16
7. C2’*....................................17
8. C4* 18

Ground-state optimized geometries

1. A

C	-3.92287500	0.89429000	0.36017800
C	-4.09756400	-0.48381500	0.16074400
C	-2.66359600	1.45982400	0.34382800
C	-1.55968700	0.63524400	0.12470500
C	-1.71118600	-0.74015300	-0.07652000
C	-3.00221600	-1.29097800	-0.05575800
O	-0.34595800	1.23364000	0.12874700
C	0.79227600	0.53023500	-0.08586200
C	0.71873900	-0.80672400	-0.32214000
C	-0.52574600	-1.54038500	-0.31261400
C	1.98348400	1.38186200	-0.06868600
C	1.84981700	2.70292600	-0.53161400
C	2.90556300	3.59387300	-0.50305800
C	4.13441500	3.17889300	0.00952700
C	4.29279700	1.88441900	0.47077700
C	3.23510500	0.97227800	0.43888600
O	-0.50034300	-2.75935500	-0.52042400
O	3.47825200	-0.24275900	0.97355700
H	2.93684600	-0.92425300	0.53371800
H	-4.78692200	1.52739700	0.53029500
H	-2.51159900	2.52189500	0.49735400
H	-5.09458400	-0.90905500	0.17702300
H	-3.10807200	-2.35828200	-0.21513700
O	1.81903800	-1.54974200	-0.59909500
H	0.88846200	3.01819900	-0.92054500
H	2.77466400	4.60350000	-0.87535100
H	4.97287000	3.86659200	0.04535100
H	5.24028500	1.54467100	0.87454100
H	1.48707600	-2.46495300	-0.68140100

2.   A’

C	-3.86880100	0.80530900	0.57275200
C	-4.01444800	-0.55278000	0.24804400
C	-2.62664800	1.40642200	0.56412200
C	-1.50978200	0.63946100	0.22700100
C	-1.63155800	-0.71490200	-0.09990700
C	-2.90764500	-1.30188100	-0.08531000
O	-0.31375600	1.26795200	0.23998300
C	0.82781200	0.60465100	-0.07447100
C	0.79807700	-0.70552700	-0.43267600
C	-0.43951200	-1.45883100	-0.46068000
C	2.01006100	1.47115900	-0.04490600
C	1.99300600	2.67990400	-0.74909900
C	3.09312600	3.52274600	-0.75818300
C	4.23292700	3.16107900	-0.04420100
C	4.26313200	1.97866000	0.67991900
C	3.15364700	1.13313800	0.69483400
O	-0.41048600	-2.64852000	-0.79933600
O	3.12426400	0.00490200	1.43670100
H	3.96004400	-0.10110600	1.90148100
H	-4.74203800	1.39317600	0.83448000
H	-2.49624600	2.45341100	0.81268400
H	-4.99851300	-1.00768700	0.26018200
H	-2.99209700	-2.35182700	-0.34298700
O	1.89835200	-1.37383600	-0.82071200
H	1.10043400	2.94300700	-1.30719500
H	3.06573600	4.44923600	-1.32025000
H	5.10481300	3.80687400	-0.04326900
H	5.14575100	1.70851400	1.25291700
H	1.57703900	-2.27002500	-1.02553200

3.   B

C	-4.01895500	0.80750700	-0.40170600

C	-4.12505000	-0.57428700	-0.18440100
C	-2.78608200	1.42984400	-0.42204900
C	-1.64398900	0.65607500	-0.22240000
C	-1.72402500	-0.72041000	-0.00469200
C	-2.98882600	-1.32887400	0.01214300
O	-0.45053700	1.30250700	-0.25504300
C	0.73364600	0.64422100	-0.08263000
C	0.73089100	-0.69841100	0.14918500
C	-0.49820500	-1.46640700	0.20439200
C	1.90032000	1.52134500	-0.16414000
C	1.85650400	2.87700100	0.23098200
C	3.00870500	3.65902500	0.15714700
C	4.19669600	3.12684500	-0.31321700
C	4.25361100	1.79636400	-0.72455400
C	3.11776200	1.01115500	-0.64568900
O	-0.42868900	-2.67948400	0.43257500
H	-4.91438800	1.39918300	-0.55800700
H	-2.68871700	2.49588500	-0.59263700
H	-5.10159600	-1.04486300	-0.17236200
H	-3.04195100	-2.39830500	0.18288000
O	1.85336600	-1.40847900	0.36171600
O	0.75544500	3.48804200	0.72215200
H	2.94340800	4.69347000	0.47651800
H	5.08016200	3.75458900	-0.36507100
H	5.17684000	1.37570000	-1.10639100
H	1.53394700	-2.31704200	0.51757900
H	3.15719200	-0.02254400	-0.96408600
H	-0.01716700	2.92801900	0.58050300

4. B’

C	-3.99225200	0.77976400	-0.66087700
C	-4.10842000	-0.56506600	-0.27455200
C	-2.76093700	1.40138600	-0.70159400

C	-1.62459500	0.66942500	-0.35108500
C	-1.71742700	-0.67170400	0.03590100
C	-2.98286200	-1.28042600	0.06977400
O	-0.44194900	1.31728600	-0.41201000
C	0.71279400	0.69616500	-0.07372300
C	0.71660200	-0.60757900	0.31172800
C	-0.50477100	-1.38196700	0.39433400
C	1.90189300	1.54600300	-0.19184300
C	1.95415300	2.81691700	0.40497200
C	3.09459500	3.60488300	0.26084400
C	4.18249300	3.13668200	-0.46213600
C	4.14940500	1.87415700	-1.04708400
C	3.01416500	1.09051300	-0.90581600
O	-0.44416300	-2.56034900	0.76713000
H	-4.88016800	1.34095900	-0.93147100
H	-2.65322900	2.43862200	-0.99725000
H	-5.08441700	-1.03637700	-0.24847500
H	-3.04398000	-2.31987600	0.37261800
O	1.84417400	-1.25395900	0.66647600
O	0.89069800	3.22481900	1.13441900
H	3.12650100	4.58497600	0.72829300
H	5.06212600	3.76361000	-0.56381500
H	4.99801100	1.50620100	-1.61226500
H	1.54210800	-2.14666700	0.91295000
H	2.97179100	0.10725900	-1.36081600
H	1.06721900	4.09439000	1.50687900

5. dep2’

C	-3.93412500	0.89670000	0.29544900
C	-4.10333400	-0.48290000	0.10228200
C	-2.67169300	1.45545700	0.31041800
C	-1.56178800	0.62543000	0.13021500
C	-1.70502800	-0.74838400	-0.06473400

C	-3.00017400	-1.29100500	-0.07565900
O	-0.35077000	1.21076200	0.16237600
C	0.79346800	0.48708400	-0.02165600
C	0.75472600	-0.87727100	-0.21883400
C	-0.51818700	-1.58504000	-0.24572800
C	1.97623800	1.34751800	-0.02498900
C	1.82532500	2.69289600	-0.41052700
C	2.88815200	3.57711800	-0.42680400
C	4.15276100	3.12154300	-0.03727800
C	4.33282700	1.80920800	0.35136800
C	3.26500700	0.87803100	0.38666900
O	-0.59470000	-2.80380800	-0.43442500
O	3.47210400	-0.33568800	0.80981400
H	-4.80110300	1.53377800	0.43513400
H	-2.51973000	2.51870200	0.45921300
H	-5.10059900	-0.90898000	0.09345300
H	-3.10122700	-2.36020500	-0.22721200
O	1.85352200	-1.59386200	-0.43964300
H	0.84111500	3.03673000	-0.70982200
H	2.74064800	4.60498500	-0.74001000
H	5.00000000	3.80181500	-0.03918300
H	5.30984200	1.45176800	0.66297300
H	2.64287100	-1.11057900	0.12072000

6. dep3

C	-3.92526900	0.89430300	0.28240300
C	-4.08835200	-0.49054000	0.11499000
C	-2.66583600	1.45796300	0.28812800
C	-1.54990400	0.63017300	0.12453100
C	-1.68673300	-0.74894100	-0.04484500
C	-2.98180700	-1.29603900	-0.04658700
O	-0.34521100	1.21970800	0.14526500
C	0.80446000	0.49059800	-0.01437000

C	0.79107800	−0.88286000	−0.19841600
C	−0.49948900	−1.58529100	−0.21158400
C	1.98297100	1.36112100	−0.01728600
C	1.83372900	2.70508100	−0.40644500
C	2.89371100	3.59536300	−0.40295200
C	4.15342800	3.15357400	0.00475800
C	4.33258400	1.83682200	0.39093800
C	3.26812700	0.92024400	0.40064300
O	−0.57716800	−2.80711200	−0.37776200
O	3.49354400	−0.31189800	0.84058300
H	−4.79521400	1.53008800	0.40932600
H	−2.51870600	2.52455000	0.41723700
H	−5.08380300	−0.92105600	0.11328000
H	−3.07851500	−2.36834000	−0.17810300
O	1.87911700	−1.57787200	−0.38983800
H	0.85266400	3.04476400	−0.71890800
H	2.74226600	4.62292500	−0.71567300
H	4.99604300	3.83828800	0.01896800
H	5.30457000	1.47831000	0.71511300
H	2.80181000	−0.97098600	0.30495400

7. 3HF

C	−3.95138700	0.89488000	0.04565000
C	−4.10462800	−0.50041000	0.01483700
C	−2.69732800	1.47128600	0.04030600
C	−1.57601200	0.64040200	0.00360200
C	−1.70615300	−0.75037700	−0.02750100
C	−2.99290100	−1.31295600	−0.02137100
O	−0.37040500	1.24496900	−0.00001200
C	0.79162600	0.54429400	−0.03424800
C	0.75039800	−0.82052600	−0.06675800
C	−0.50182200	−1.55227800	−0.06525800
C	1.96626600	1.41772600	−0.03228200

C	1.78925500	2.81078000	-0.01695500
C	2.88319500	3.66293100	-0.01438200
C	4.17602400	3.14778100	-0.02618300
C	4.36276400	1.76984300	-0.04083700
C	3.27411300	0.90783900	-0.04418600
O	-0.46102300	-2.78945500	-0.09639400
H	3.43918900	-0.15999600	-0.05534200
H	-4.82857200	1.53223400	0.07408900
H	-2.56197000	2.54643400	0.06381600
H	-5.09825600	-0.93395900	0.01963300
H	-3.08197300	-2.39334000	-0.04573900
O	1.84624800	-1.59999000	-0.10353400
H	0.78880800	3.22439200	-0.00738500
H	2.72408900	4.73608400	-0.00296300
H	5.03091700	3.81579200	-0.02395700
H	5.36613600	1.35713800	-0.04972100
H	1.48635900	-2.50811900	-0.12051500

8. C2’

C	-3.93326400	0.79968800	0.45242900
C	-4.06797700	-0.57087800	0.16202000
C	-2.69978500	1.41476600	0.44299400
C	-1.58247700	0.64165500	0.13708400
C	-1.69118900	-0.72562100	-0.15435500
C	-2.96407000	-1.33073500	-0.13823400
O	-0.38569400	1.26014700	0.12264400
C	0.75965400	0.62454800	-0.13463300
C	0.73924500	-0.72933100	-0.43083200
C	-0.48963300	-1.40965500	-0.44968400
C	1.92243600	1.49104800	0.00400300
C	1.87880800	2.51578200	0.96587500
C	2.95055800	3.36576200	1.15392300
C	4.09150600	3.21875700	0.36675500

C	4.15413100	2.22922600	-0.60083200
C	3.07631700	1.37307600	-0.78880700
O	-0.51590100	-2.68942700	-0.71684900
O	3.08883200	0.42691200	-1.76666400
H	-4.81519500	1.38455100	0.68782800
H	-2.57805700	2.46827100	0.66310100
H	-5.05091900	-1.02653900	0.17720800
H	-3.04933500	-2.38706300	-0.36208600
O	1.81510800	-1.50823400	-0.62872600
H	0.99101900	2.61786100	1.57830100
H	2.90267000	4.13802400	1.91226000
H	4.93886300	3.88126000	0.50394500
H	5.03431800	2.12298700	-1.22687100
H	0.39097800	-2.99029700	-0.90198400
H	3.91108200	0.45684800	-2.26992700
H	2.49420800	-1.01022600	-1.13246600

9. C4

C	-3.94637600	0.85642500	0.52473500
C	-4.11910100	-0.49859900	0.19127600
C	-2.69557700	1.43395000	0.52269500
C	-1.60361900	0.64157800	0.17980600
C	-1.74658100	-0.71362900	-0.15877300
C	-3.03884100	-1.27689200	-0.14566400
O	-0.39512600	1.23510400	0.17252600
C	0.73502600	0.59640000	-0.12294700
C	0.67379900	-0.75436500	-0.43807400
C	-0.55618100	-1.41455800	-0.47439200
C	1.91180400	1.43993300	-0.00878300
C	1.85641200	2.47193500	0.95443500
C	2.88828400	3.36863000	1.11446200
C	4.01108700	3.27054600	0.28863200
C	4.09078800	2.27631300	-0.66596900

C	3.06144100	1.34555800	-0.83168000
O	-0.48314900	-2.69127300	-0.78395100
O	3.22305800	0.47122900	-1.83737300
H	-4.80975500	1.45699200	0.78723400
H	-2.53937800	2.47561000	0.77507500
H	-5.11213300	-0.93156000	0.20031200
H	-3.19330800	-2.31993900	-0.39836900
O	1.81870600	-1.42938000	-0.67081700
H	0.98132500	2.54008200	1.58947400
H	2.82648600	4.13832600	1.87457900
H	4.83020200	3.97356200	0.39672400
H	4.95349200	2.19022600	-1.31676400
H	-1.33488400	-3.14541700	-0.78299600
H	2.74329500	-0.35555800	-1.66127500
H	1.62770600	-2.35203200	-0.89175300

Excited-state optimized geometries

10. A*

C	−3. 94396700	0. 93474800	−0. 00351200
C	−4. 13014400	−0. 44661600	−0. 00726500
C	−2. 65461600	1. 47472500	−0. 00225600
C	−1. 56367700	0. 62528100	−0. 00485100
C	−1. 72595400	−0. 77472900	−0. 00886200
C	−3. 03438200	−1. 29547300	−0. 00991600
O	−0. 33281100	1. 22141600	−0. 00322900
C	0. 84446500	0. 51165100	−0. 00538900
C	0. 70565500	−0. 87862800	−0. 01148100
C	−0. 55108800	−1. 58440500	−0. 01230900
C	1. 99294200	1. 36245200	−0. 00187300
C	1. 77504300	2. 76581100	−0. 00133500
C	2. 80838200	3. 67814600	0. 00321800
C	4. 14892200	3. 24941200	0. 00802600
C	4. 41773800	1. 89877700	0. 00804500
C	3. 38938100	0. 94518900	0. 00303500
O	−0. 47195900	−2. 85115400	−0. 01650500
O	3. 80329500	−0. 30714400	0. 00400100
H	3. 07516200	−0. 97228000	−0. 00167000
H	−4. 79916500	1. 60154000	−0. 00151900
H	−2. 49173600	2. 54654000	0. 00072200
H	−5. 13374600	−0. 85798700	−0. 00817400
H	−3. 15776400	−2. 37260900	−0. 01293900
O	1. 72869900	−1. 72964400	−0. 01720300
H	0. 75534400	3. 12265400	−0. 00482300
H	2. 57969800	4. 73812600	0. 00306900
H	4. 95855300	3. 96999300	0. 01164600
H	5. 43373500	1. 52060200	0. 01182800
H	1. 25333700	−2. 61710800	−0. 01745700

11. B*

C	-4.04584000	0.82722700	-0.08746900
C	-4.15045700	-0.55907100	-0.03146000
C	-2.78352200	1.43820700	-0.12713300
C	-1.65553800	0.64753500	-0.11089600
C	-1.72839400	-0.75708200	-0.05642800
C	-3.00887300	-1.34760300	-0.01534100
O	-0.44111200	1.30327100	-0.15815000
C	0.77826000	0.66046200	-0.12906200
C	0.72434000	-0.74199700	-0.07528000
C	-0.51607500	-1.50602600	-0.04072000
C	1.91840100	1.50314000	-0.15633900
C	1.82098500	2.95249900	-0.10912300
C	2.96708900	3.74682000	-0.13276100
C	4.22253300	3.17743600	-0.20186500
C	4.34755900	1.77556000	-0.25156800
C	3.23672900	0.96728800	-0.23053800
O	-0.38684300	-2.76203700	0.01078900
H	-4.93571300	1.44612100	-0.10078600
H	-2.68719300	2.51712000	-0.17232600
H	-5.12873900	-1.02662100	-0.00022700
H	-3.07338900	-2.42871100	0.02893000
O	1.79098100	-1.51138800	-0.03613600
O	0.66180100	3.60013200	-0.02762600
H	2.83161300	4.82182400	-0.09297400
H	5.10493500	3.80706400	-0.22023500
H	5.33137000	1.32392500	-0.31268200
H	1.38132800	-2.41992600	0.00158200
H	3.36240100	-0.10377600	-0.27592600
H	-0.06831700	2.95627600	-0.03955400

12. T2’*

C	-3.92716100	0.72328300	0.75757100

C	-4.11017000	-0.58817700	0.30267400
C	-2.66282800	1.30154000	0.75878300
C	-1.58115200	0.56259200	0.30290500
C	-1.74130700	-0.76147600	-0.16055300
C	-3.03578600	-1.32668800	-0.15184200
O	-0.37004800	1.15894300	0.31985400
C	0.77889500	0.53359200	-0.10555200
C	0.67679200	-0.77620500	-0.57360200
C	-0.58269700	-1.42241300	-0.60062700
C	1.93482100	1.41598600	0.02665900
C	1.74793800	2.69898100	0.53377800
C	2.80386600	3.60773500	0.69249700
C	4.10467700	3.25071700	0.34070500
C	4.33714100	1.98702900	-0.16690100
C	3.29270400	1.03954200	-0.34186300
O	-0.65346500	-2.67629900	-1.05319500
O	3.59114500	-0.11265300	-0.81918800
H	2.57923500	-0.94683000	-0.95758100
H	-4.77566900	1.29728100	1.11308600
H	-2.50170600	2.31528600	1.10711100
H	-5.10221600	-1.02617300	0.30756200
H	-3.16458700	-2.34250100	-0.50639000
O	1.67414400	-1.51997800	-1.01732000
H	0.75135000	3.00994600	0.81716200
H	2.59448600	4.59330000	1.09315200
H	4.92189700	3.95285300	0.46306500
H	5.33235800	1.66518100	-0.45409400
H	0.24679200	-2.94093200	-1.30300800

13. T3*

C	-3.90429700	0.70151100	0.82025600
C	-4.09376300	-0.58620000	0.31008500
C	-2.64038900	1.29014800	0.81303600

C	-1.56922400	0.58497600	0.29372200
C	-1.73494400	-0.71773100	-0.22772200
C	-3.02618700	-1.29311600	-0.20925500
O	-0.36107100	1.19778300	0.30806500
C	0.78843200	0.61591300	-0.17416700
C	0.68129600	-0.69688900	-0.71211500
C	-0.58717400	-1.34695300	-0.73036400
C	1.92333400	1.47543000	-0.03885700
C	1.73408100	2.75551500	0.54578300
C	2.76852700	3.65148200	0.71731900
C	4.07092800	3.32097600	0.31102500
C	4.30677000	2.08901800	-0.26271900
C	3.27695600	1.15432000	-0.45182100
O	-0.64679900	-2.57465400	-1.24165900
O	3.61898200	0.01491100	-1.00798100
H	-4.74447700	1.25347500	1.22710000
H	-2.47807700	2.28760400	1.20501700
H	-5.08230700	-1.03168600	0.32209300
H	-3.15746700	-2.29215000	-0.60826700
O	1.65930500	-1.37508300	-1.20106800
H	0.73881300	3.03062900	0.86591300
H	2.56819100	4.61663200	1.16984400
H	4.88483300	4.02506000	0.44571200
H	5.29904900	1.79741500	-0.58932700
H	0.26091000	-2.79354600	-1.52032000
H	2.80889900	-0.63316200	-1.11918800

14. RT3*

C	-3.88961100	1.05492800	0.12557000
C	-4.13182500	-0.31356800	0.01348900
C	-2.57802900	1.54506000	0.13952600
C	-1.52922800	0.65670300	0.04101500
C	-1.74296600	-0.73452400	-0.07234600

C	-3.07763100	-1.20507800	-0.08490000
O	-0.26201300	1.17613800	0.05968200
C	0.88890800	0.41970100	-0.03057300
C	0.72847600	-0.99980800	-0.15234300
C	-0.60721200	-1.54537900	-0.16586700
C	2.09949600	1.15813000	0.01335400
C	3.35905600	0.48721900	0.01156100
C	4.55110200	1.17036700	0.05181900
C	4.56589900	2.57251700	0.09862400
C	3.36928000	3.26635800	0.10394400
C	2.14760300	2.59954000	0.06231500
O	-0.70372800	-2.86449800	-0.28157300
O	1.05831500	3.38288400	0.05857500
H	-4.71620000	1.75210800	0.20287200
H	-2.38037000	2.60720400	0.22763100
H	-5.15150000	-0.68237200	0.00322200
H	-3.25298400	-2.27084900	-0.17254400
O	1.67139000	-1.83425500	-0.25705100
H	3.35267700	-0.59264000	-0.02222700
H	5.48349900	0.61632900	0.05169300
H	5.50547300	3.11333300	0.13265100
H	3.34629900	4.35028800	0.13881100
H	0.22184400	-3.17832100	-0.33232000
H	0.26602900	2.82510100	0.04893100

15. dep*

C	-3.92881900	0.94433600	-0.01764600
C	-4.12377700	-0.43270700	0.01584800
C	-2.62744400	1.46851900	-0.01775000
C	-1.54804200	0.60225200	0.01572700
C	-1.71465500	-0.79011700	0.04990800
C	-3.03246100	-1.29386100	0.04929400
O	-0.31052600	1.17329200	0.01383400

C	0.85670600	0.45371300	0.04403900
C	0.75837400	-0.97564600	0.07996800
C	-0.55699100	-1.64960700	0.08399300
C	2.00060000	1.31102800	0.03345300
C	1.77620200	2.72605500	-0.00507400
C	2.79969400	3.64560600	-0.01830300
C	4.13540100	3.21913500	0.00616900
C	4.40520300	1.86140600	0.04372300
C	3.38853700	0.89953500	0.05806400
O	-0.63738400	-2.89938300	0.11531700
O	3.77854900	-0.36322700	0.09427900
H	-4.77712500	1.61981500	-0.04381700
H	-2.44844900	2.53773900	-0.04347900
H	-5.13101900	-0.83728700	0.01589400
H	-3.16465400	-2.36956700	0.07565900
O	1.79198300	-1.72357500	0.10951400
H	0.75427200	3.07792500	-0.02439200
H	2.56485000	4.70474900	-0.04781700
H	4.94799800	3.93797900	-0.00398800
H	5.42574700	1.49274800	0.06351900
H	2.95131900	-0.99791600	0.10240900

16. C2'*

C	-3.87837900	0.90832100	0.37547400
C	-4.07757700	-0.44824400	0.10091800
C	-2.59531300	1.45814700	0.37274000
C	-1.51889200	0.64105200	0.09254900
C	-1.69149800	-0.72969700	-0.18781100
C	-3.00044000	-1.26480800	-0.17772100
O	-0.28868500	1.21333500	0.10167900
C	0.87052900	0.53133100	-0.15502300
C	0.74022100	-0.85325800	-0.43386000
C	-0.53432600	-1.47798600	-0.45749000

C	1.99599200	1.42265700	-0.14745100
C	1.73902800	2.81471700	-0.03311200
C	2.74945300	3.74939700	-0.00697900
C	4.09585200	3.35476100	-0.08489700
C	4.40154900	2.01152600	-0.18850200
C	3.39035700	1.06212800	-0.22003300
O	-0.63519700	-2.77731400	-0.72174700
O	3.70793000	-0.24133600	-0.29915500
H	-4.72652800	1.54715800	0.59458700
H	-2.42840700	2.50754100	0.58465200
H	-5.08048700	-0.85887500	0.10783200
H	-3.13471200	-2.31826600	-0.39178900
O	1.71797000	-1.69366900	-0.70958000
H	0.71270500	3.14509800	0.02566000
H	2.49931300	4.80091800	0.07284500
H	4.88971900	4.09208900	-0.06298000
H	5.43357500	1.67976800	-0.24240900
H	0.24699600	-3.14636800	-0.87396400
H	4.66157200	-0.39181600	-0.35290900
H	2.60680200	-1.27399700	-0.61085300

17. C4*

C	-3.71342400	0.29650800	1.49861900
C	-4.00515600	-0.78657400	0.66665300
C	-2.46246900	0.89934100	1.45122700
C	-1.50793900	0.40843900	0.57674500
C	-1.77350000	-0.68914900	-0.27201900
C	-3.05305400	-1.27373600	-0.20966400
O	-0.30377100	1.04011000	0.56532900
C	0.70234600	0.64809000	-0.26996800
C	0.49752500	-0.42849700	-1.09641900
C	-0.72540900	-1.11174800	-1.12365900
C	1.91488800	1.45716400	-0.05875700

C	2.10625300	2.04108300	1.17361400
C	3.18096400	2.92734400	1.43794400
C	4.10007500	3.25200800	0.44423400
C	3.94871100	2.68099200	-0.79887900
C	2.88977600	1.76734600	-1.06432100
O	-0.78877500	-2.12144200	-2.00949200
O	2.89147400	1.26325600	-2.26971600
H	-4.46350600	0.67428900	2.18365900
H	-2.21391900	1.74502000	2.08169900
H	-4.98476900	-1.24907900	0.70359200
H	-3.30562700	-2.11034300	-0.85266100
O	1.49939200	-0.84162700	-1.91543800
H	1.39893400	1.83803500	1.96743900
H	3.26339800	3.36410200	2.42589800
H	4.91208000	3.94014700	0.64123100
H	4.62884400	2.89024600	-1.61608000
H	-1.59663500	-2.63714700	-1.92372200
H	2.30461700	0.46712800	-2.34887500
H	1.18668400	-1.55993500	-2.48454200
